# A call for objectivity: Radiologists’ proposed wishlist for response evaluation in solid tumors (RECIST 1.1)

**DOI:** 10.1186/s40644-024-00802-8

**Published:** 2024-11-14

**Authors:** Kathleen Ruchalski, Jordan M. Anaokar, Matthias R. Benz, Rohit Dewan, Michael L. Douek, Jonathan G. Goldin

**Affiliations:** 1 Department of Radiological Sciences, University of California Los Angeles, Los Angeles, USA; 2 Ahmanson Translational Theranostics Division, Department of Molecular and Medical Pharmacology, University of California Los Angeles, Los Angeles, USA; 3 1250 16th Street, Suite 2340, Santa Monica, CA 90404 USA

**Keywords:** RECIST, Progression free survival., Disease progression, Biomarkers, Tumor, Clinical trial

## Abstract

The Response Evaluation in Solid Tumors (RECIST) 1.1 provides key guidance for performing imaging response assessment and defines image-based outcome metrics in oncology clinical trials, including progression free survival. In this framework, tumors identified on imaging are designated as either target lesions, non-target disease or new lesions and a structured categorical response is assigned at each imaging time point. While RECIST provides definitions for these categories, it specifically and objectively defines only the target disease. Predefined thresholds of size change provide unbiased metrics for determining objective response and disease progression of the target lesions. However, worsening of non-target disease or emergence of new lesions is given the same importance in determining disease progression despite these being qualitatively assessed and less rigorously defined. The subjective assessment of non-target and new disease contributes to reader variability, which can impact the quality of image interpretation and even the determination of progression free survival. The RECIST Working Group has made significant efforts in developing RECIST 1.1 beyond its initial publication, particularly in its application to targeted agents and immunotherapy. A review of the literature highlights that the Working Group has occasionally employed or adopted objective measures for assessing non-target and new lesions in their evaluation of RECIST-based outcome measures. Perhaps a prospective evaluation of these more objective definitions for non-target and new lesions within the framework of RECIST 1.1 might improve reader interpretation. Ideally, these changes could also better align with clinically meaningful outcome measures of patient survival or quality of life.

Response Evaluation in Solid Tumors (RECIST) 1.1 is the most commonly used imaging response assessment criteria when evaluating treatment response in oncology. This consensus publication provides guidelines for the standardization of image acquisition, radiologic interpretation, and image-based outcome measures to be included in oncology therapeutic trials [1]. Increasingly, RECIST 1.1-based outcome measures, most commonly progression free survival (PFS), have been used as primary outcome measures for regulatory drug approval [2]. However, there has been growing discussion questioning if PFS alone is an appropriate intermediate endpoint worthy of regulatory approval [3, 4]. In addition to mounting evidence challenging the validity of PFS as a surrogate biomarker for overall survival, there are also known inherent limitations in the measurement of PFS. Although certain constraints, like scheduling of image evaluations and patient censoring, extend beyond the realm of image analysis, inaccuracies in tumor measurements and interpretation of RECIST 1.1 guidelines may also play a role [3]. Limitations in RECIST 1.1 assessments are well described and some degree of reader variability is expected. Radiological clinical experience, clinical trial experience as well as reader training aid in reducing inter-reader variability [5]. Blinded independent review committees with a dual reader adjudication paradigm are also commonly used in registered trials to offset these errors and improve reliability in radiologic data interpretation [5].

RECIST 1.1 was published in the era of cytotoxic therapies and later reassessed to show similar validity for cytostatic agents [6]. There have also been multiple modifications for treatment assessment in immunotherapy, including irRECIST, imRECIST and iRECIST [7–9]. Yet the fundamentals of RECIST have remained the same since 2009. Readers measure up to five target lesions to represent a quantitative surrogate for total tumor burden. All other lesions, including measurable disease beyond the five targets, truly non-measurable disease and new lesions on follow up studies are only qualitatively assessed [1] (Table 1). While this may not have as much an impact on assessing objective response rates, it certainly lessens objectivity in the evaluation for PFS, as progression by non-target lesions alone and/or the development of new lesions can define progressive disease (PD) [1, 10]. Moreover, these subjectively evaluated lesions carry critical prognostic value. Data from a retrospective review of the RECIST warehouse indicate that appearance of new lesions (HR = 2.14 (95% CI 1.97–2.33)) or progression by non-target disease (HR = 1.65 (95% CI 1.51–1.80)) correlates with reduced overall survival compared to an increase in measured tumor load (HR = 1.02 (95% CI 1.02–1.03) [6, 11].

**Table 1:** RECIST 1.1 time point response

Target lesions	Non-target lesions	New lesions	Overall Response
CR	CR	No	CR
CR	Non-CR/non-PD	No	PR
CR	Not evaluated	No	PR
PR	Non-PD or not all evaluated	No	PR
SD	Non-PD or not all evaluated	No	SD
Not all evaluated	Non-PD	No	NE
PD	Any	Yes or No	PD
Any	PD	Yes or No	PD
Any	Any	Yes	PD

CR = complete response, PR = partial response, SD = stable disease, PD = progressive disease, and NE = inevaluable [1]

For a radiologist, basing the decision of “PD versus not PD” solely on the measurements of a maximum of five tumors is particularly challenging in cases with a high tumor burden. If there were to be a future revision of RECIST, as radiologists interpreting these studies, we would propose more objectivity in the definitions and assessment of each component of RECIST 1.1 (Table 2). Providing more guidance in these areas may prove to be beneficial for a few reasons. First, reducing the subjectivity of these assessments may improve reader agreement and enhance the quality of radiologic interpretation of PFS and secondly, potentially align RECIST -based outcomes more closely with meaningful clinical outcomes of patient survival and quality of life.

**Table 2:** Wishlist for clarifications to RECIST 1.1

RECIST 1.1 Features	Challenges/Questions	Potential solution to investigate/Clarifications requested
*Target Lesions*	Variability in baseline target lesion selection as major reason for inter-reader variability	Restore number of allowable target lesions to 10 which may reduce variability in target lesion selection and encourage readers to choose more targets.
	Heterogeneity in response/underrepresentation of tumor response per organ	Allow for more than two targets per organ to better reflect distribution of disease when it disproportionally affects one or more organs, as allowable per PCWG3
*Non-Target Lesions*	Can progression of measurable non-target disease occur based on growth of a single or few lesions, grouped lesions by organ (i.e. progression in liver), or only as a global assessment of all non-target disease?	Clarify if trial readers may apply the same parameters used in the study validating RECIST 1.1 for targeted agents, which considered non-target lesions to have progressed if they have doubled in size Clarify if non-target assessment is at individual lesion level or grouped/total tumor burden
	RECIST 1.1 recommended threshold of a 73% in the volume of non-measurable disease, meant to be comparable to a 20% increase in SOD, is difficult to confidently visually assess.	Allow for an easier threshold to assess, such as 100% change (doubling)
*New Lesions*	Development of new lesions is a common cause of adjudication and there is no minimum size threshold prescribed for new lesions. When can a lymph node that is present at baseline and subsequently enlarges be considered new?	Use same thresholds for target lesions, or require confirmation with growth on at least one follow up time point for suspected new lesions under this threshold measurement. Require minimum 15 mm short axis dimension for new lymph nodes, which would also satisfy the requirement of at least 5 mm absolute increased in size for PD.
*Special Cases: Bone*	How should previously lytic lesions with a measurable soft tissue component used as a target be measured when the soft tissue component has re-mineralized? How should lytic non-target lesions that have become sclerotic as part of healing, or sclerotic lesions that show no metabolic activity on bone scan/PET CT be assessed?	Clarification that only soft tissue components should be included in target lesion measurement, even if a sclerotic or remineralized remnant of the lesion remains. When PETCT studies are available, lack of activity associated with these lesions can be considered CR. In the absence of correlative metabolic studies, these can considered Non-CR/Non-PD.
*Special Cases: Pleura*	Measurements of pleural lesions can be difficult to reproduce, and carcinomatosis is often unmeasurable.	Clarify if the same principles as mRECIST for mesothelioma (measuring perpendicular thickness) can be used to more objectively assess pleural disease.
*Special Cases: Free fluid*	Pleural effusions and abdominal ascites may not be malignant in etiology or be a reliable indicator for change in disease status	Do not take pleural effusions and ascites into account for response assessment. Only evaluate changes in soft tissue components in present in accompanying pleural or peritoneal carcinomatosis.
*Special Cases: PET-CT*	Incorporating findings from standard of care PET-CT if performed prior to trial entry. How should site readers consider metabolically active lymph nodes suspected to be malignant but do not meet RECIST 1.1 size requirements as pathologic?	Allow metabolically active lymph nodes suspected to be metastatic to be considered non-target disease, even if they fall below < 10 mm short axis minimum threshold.

## Target lesions

Target lesion measurements represent the only objective assessment in RECIST 1.1. The assumption is that while individual target lesions may shrink or grow to varying degrees, RECIST 1.1 captures the global response at the patient level by giving a single categorical response at each timepoint. According to RECIST 1.1; up to five target lesions; two per organ can be measured on a patient’s baseline imaging and then serially followed and re-assessed for size change while on treatment [1]. This was reduced from what was originally defined in RECIST 1.0; where up to ten lesions total, five lesions per organ was the recommendation [1, 12]. To support this guideline update, the RECIST Working Group had performed retrospective studies demonstrating high levels of reader agreement and no significant differences in PFS when reducing the number of target lesions to five [13, 14]. Using pooled trial data, Bogaerts et al. demonstrated that changing the number of target lesions to only five in version 1.1 resulted in only a 0.4% change in number of PFS events [15]. Moskowitz et al. also utilized simulated data to evaluate a change from ten target lesions to five lesions and found that shifting the number of target lesions from 10 to five resulted in only a change in 2% of patients being classified as progressors versus non-progressors. However this experiment evaluated radiologists selecting the *same* up to ten lesions [14]. A more recent prospective reader study demonstrated that which lesions are selected as targets matters. In this study by Kuhl et al., there was high reader agreement for categorical response and disease progression when the same target lesions were selected amongst readers (k = 0.97; 95% confidence interval (CI): 0.91, 1.0 and k = 0.98; 95% CI: 0.90, 1.0 respectively) but significantly decreased reader agreement when target lesion selection differed (k = 0.58; 95% CI: 0.54, 0.62 and k = 0.6; 95% CI: 0.59, 0.70) [16]. Variability in baseline disease selection is known to be one of the major contributors to RECIST 1.1 inter-reader variability [17]. This can be even more problematic in patients with high tumor burden where reader discordance may increase as the total number of tumors available to select as five target lesions per patient increases [18].

**Fig. 1:**
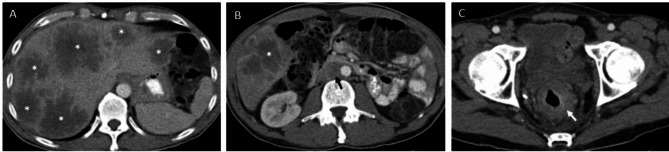
Target lesion: Single organ dominant disease

Selecting the same target lesions, or potentially few target lesions, in RECIST 1.1 may underscore inter-tumor biologic heterogeneity and thus under-recognize inherent differences in target lesion response [16]. Prostate cancer working group 3 (PCWG3) has already integrated concepts of tumor heterogeneity and mixed response into their recommendations for prostate cancer clinical trials. While RECIST 1.1 conceptually remains the backbone for imaging response assessment of soft tissue disease by this guideline, PCWG3 recommends measuring and serially following up to ten targets, including up to five lesions per site of disease spread (e.g. liver, lung, lymph node) to account for tumor heterogeneity [19]. In comparison, RECIST 1.1 allows for only up to two target lesions per organ [1]. This encourages targets to be selected from all sites of disease spread. However, certain tumor types have a predilection to metastasize to specific organs, leading to occasions in which a disproportionate amount of disease resides in only one organ. In these instances, measuring only two lesions may significantly underrepresent the amount of total tumor burden in a patient (Fig. 1). This may also lead to an underrepresentation of important predictive and/or prognostic information regarding treatment outcomes. For example, it is known that liver metastases in melanoma and non-small cell lung cancer treated with pembrolizumab are associated with reduced responses and PFS [20].

Perhaps allowing more lesions to become measurable disease may not only improve reader agreement but also not be as burdensome as first anticipated [21]. In multiple prior studies, only three target lesions at most were measured either on average or the majority of the time when RECIST 1.1 was used [13, 16, 17, 21, 22]. Allowing more targets per organ site could bolster the total measurable disease burden closer to even RECIST 1.1 recommendations of five lesions total.

*A 54 year old man with metastatic rectal cancer with liver dominant disease on pre-treatment imaging. Multiple large hypoenhancing liver lesions were noted throughout the liver (A). A few nodal metastases were also present in the abdomen and pelvis; most of which did not meet size criteria for target disease by RECIST 1.1 (B). A retroperitoneal lymph node (black arrow) measures 16 mm in short axis and may be selected as a target lesion*, but is much smaller in size than the adjacent liver metastasis (*),* which measures 46 mm. Non-measurable disease was also present*,* including the primary neoplasm (C) represented by irregular rectal wall thickening (white arrow) and few sclerotic bone lesions (not included).*

### Redefining non-target disease progression

Non-target disease progression as originally defined in RECIST 1.1 is subjective and vulnerable to bias and inter-reader variability [22, 23]. Litiere et al. from the RECIST working group has even acknowledged that non-target lesion progression is variably defined and variably implemented [11]. According to RECIST 1.1, non-target only disease progression requires, “an overall level of substantial worsening in non-target disease, such that even in the presence of SD or PR in target disease, the overall tumor burden has increased sufficiently to merit discontinuation of therapy” [1]. This concept of subjective growth sufficient to discontinue therapy inherently introduces clinical assessment and bias into image interpretation. Imaging is just one component of the clinical picture, so what is to be done when radiographic non-target progression is not matched with the same degree of clinical progression? In addition, a non-blinded radiologists may have access to the clinical status of the patient and be sensitive to the availability or lack of subsequent therapy options at the point of determining progression. These considerations create a loss of objectivity in image assessment that can distort PFS.

Although RECIST 1.1 describes PD based solely on unequivocal worsening of non-target disease to be a rarity, several studies have subsequently found it to represent a substantial minority of cases. A retrospective review of RECIST warehouse data found PD by only non-target disease progression in 28% of patients [11]. An additional study found non-target progression as a source of PD for 19% of patients with metastatic renal cell carcinoma [24]. In both cases, non-target only progression was associated with decreased PFS and OS (HR = 1.5- 2.0) [11, 24].

**Fig. 2:**
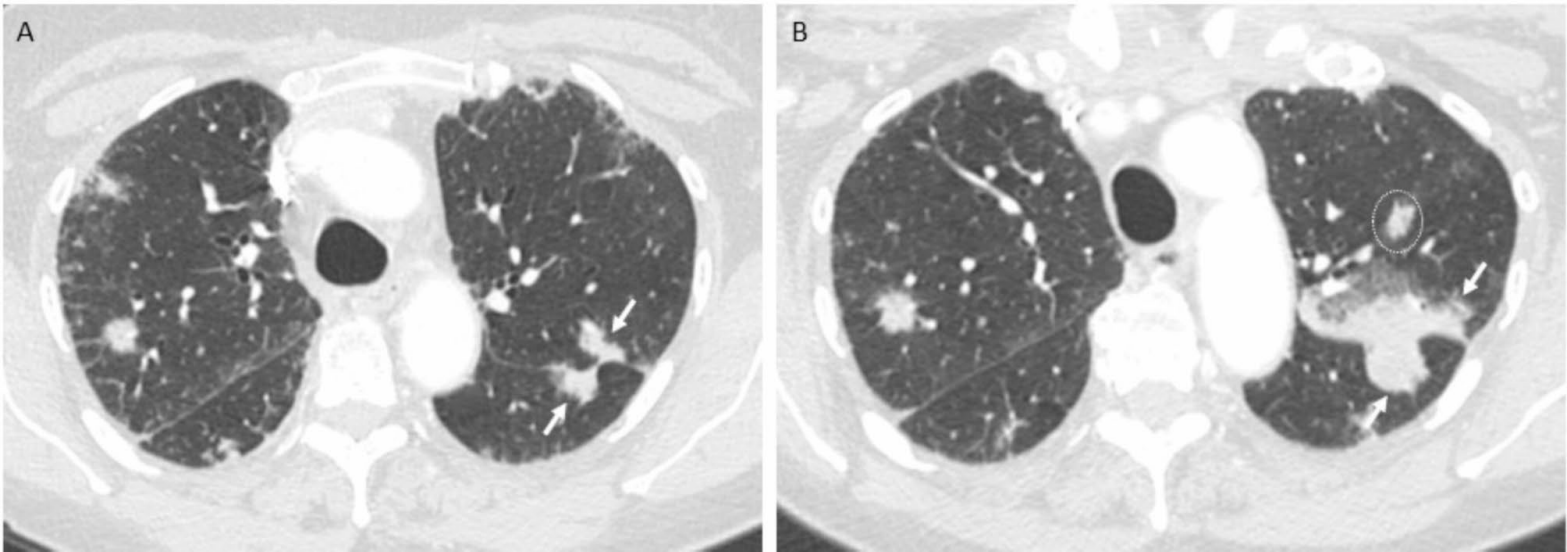
Non-target progressive disease

An updated definition of non-target progression with quantifiable guidelines could help minimize the subjectivity and variability in assessing non-target disease. The current guidance when assessing progression of non-measurable disease is to consider if the increase would be comparable in magnitude to the increase required to declare PD for measurable disease. For example, an increase in the tumor “volume” by 73% is equivalent to the 20% increase in a target lesion [1]. Yet, a 73% change in volume is a challenging threshold to confidently assess with eyes alone for something that is by definition non-measurable. Additionally, for non-target disease that could be measured, RECIST 1.1 does not explicitly define if unequivocal PD by non-target disease alone can be a result of individual lesions, grouped lesions at the organ level or as an overall non-target assessment [1](Fig. 2). Interestingly, the RECIST working group included new parameters for non-target disease progression during their validation of RECIST 1.1 for treatment with targeted agents [6]. For this analysis, non-target pathologic lymph nodes (between 10 and 15 mm short axis) were considered to represent progression if they doubled in size (+ 100% growth). Similarly, measured lesions not selected as targets were considered to have progressed when doubled in size [6]. While originally for the purposes of retrospective comparison, an evaluation of these new, albeit conservative growth thresholds could provide more objective criteria for non-target progression, thus removing the subjectivity and variability in non-target assessment. After all, it was in this context that non-target disease progression held prognosis for decreased overall survival [6]. Of course, there will always be exceptions to the rule, where objectively quantifying a doubling in size of truly non-measurable disease, such as lymphangitic carcinomatosis or peritoneal carcinomatosis will be limited and where a cancer imager’s experience and training are essential.

*A 73 year old female patient with pancreatic adenocarcinoma metastatic to the lungs has target and non-target pulmonary metastases at baseline (A). On 18 week follow up (B)*,* the tumor burden overall increased*,* but not to the point of target progression (SOD + 17% from nadir). Non-target tumor along the left major fissure (arrows) disproportionately increased relative to the other lesions and more than doubled in size*,* but it is unclear in the current guidelines if this constitutes disease progression. An additional left upper lobe nodule (circle) on follow up was present at baseline*,* but not included on the displayed image.*

## Definition of new lesions

**Fig. 3:**
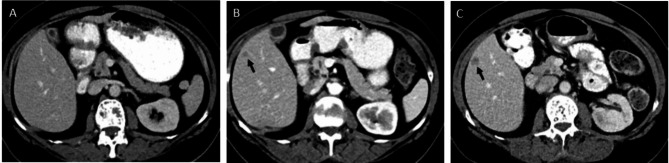
Development of a new lesion

The development of new metastatic lesion(s) can alone define PD in RECIST 1.1 [1]. However, what constitutes a new lesion is only loosely defined. According to RECIST 1.1: “There are no specific criteria for the identification of new radiographic lesions; however the findings of a new lesion should be unequivocal [1].” New lesions must be first visualized, then identified as new and finally determined to be metastatic. Even if small new lesions are seen, readers may choose to wait to determine if a lesion grows and proves to be malignant before calling it a definite new lesion [5] (Fig. 3). Although retroactively revising timepoint assessments to reflect PD to when the lesion initially appeared is prescribed in RECIST 1.1, this is likely inconsistently done [1]. Given reader variability may occur in any of these steps, development of a new lesion is a common cause for adjudication and can be the cause for up to 50% of cases of reader discordance in oncology clinical trials [22, 25, 26].

*A 62 year old woman with metastatic breast cancer enrolled in a clinical trial. No liver disease was present at baseline (A). A new 7 mm hypodense lesion was noted in the liver at week 40 (B) and was deemed suspicious but not definite for new lesion by RECIST 1.1. At week 48 (C) this lesion increased in size to 14 mm and was classified as disease progression. A subsequent liver biopsy confirmed metastatic breast cancer.*

In addition to improving reader agreement, could size thresholds help determine what is a clinically significant new lesion? While RECIST 1.1 does not have a size requirement, more recent modifications of RECIST for immunotherapy include size thresholds for new measurable lesions. Both, irRECIST and iRECIST require measurable new lesions to meet the same criteria as used for target lesions at baseline ($\:\ge\:$10 mm for solid lesions; $\:\ge\:$15 mm short axis for lymph nodes) [7, 9]. Perhaps including these metrics into a RECIST 1.1 revision could result in a more cohesive interpretation of new lesion development and allow for further objective and prospective investigation as to the prognostic value of new lesions.

While PETCT has become more widely available and is readily used in standard of care response assessment in oncology, it is less commonly incorporated into the clinical trial setting when evaluating solid tumors. PETCT is the primary imaging modality for lymphoma, and is regularly included into clinical trial design for response assessment in this setting [27]. Interim PETCT has even been used in numerous clinical trials for Hodgkins Lymphoma to incorporate risk-adapted treatment regimens based in imaging findings [28]. Yet, imaging response assessment of solid tumors is predominately reliant on CT and MRI evaluation. Even without incorporating PET Response Criteria in Solid Tumors (PERCIST), utilization of PETCT at baseline and response assessment could help provide better evaluation of tumor response in certain scenarios. Incorporation of FDG-PETCT at baseline aids in selecting target lesions by utilizing the presence of metabolic activity to prioritize the CT measurement of viable tumor only [29]. On follow up imaging, incorporating metabolic activity could allow for more objective assessment of non-target disease, including bone disease, in which RECIST 1.1 is problematic in assessing tumor viability when soft tissue is not present. The recent rapid growth in available radiotracers is continually improving our understanding of tumor biology and metastatic spread. For example, “next generation imaging” with PSMA PETCT has improved the detection of prostate cancer metastases and can more readily detect nodal metastases that are not considered pathologic by RECIST 1.1 criteria [30].

## Conclusion

RECIST 1.1 has been thoughtfully crafted, widely implemented and is considered the gold standard in tumor response assessment. The RECIST Working Group has continued to explore further variations and alternatives to the current version, including alternate categorical cut points and continuous tumor-based metrics, none of which have been found to be superior to the ease of simply applying RECIST 1.1 [15, 31]. While exploring other imaging modalities and metrics of tumor response are necessary, we believe that there is growing evidence that evaluation and modifications of the main components could be beneficial.

Ongoing advances in medical imaging, artificial intelligence and computational power will also assist with RECIST 1.1 evolution in the future. This could make widespread availability of additional imaging metrics, including volumetric assessment of individual and total tumor burden, which may also improve the use of imaging response assessment for oncology clinical trials.

## Data Availability

No datasets were generated or analysed during the current study.
